# PharmActa: Empowering Patients to Avoid Clinical Significant Drug–Herb Interactions

**DOI:** 10.3390/medicines6010026

**Published:** 2019-02-16

**Authors:** Marios Spanakis, Stelios Sfakianakis, Vangelis Sakkalis, Emmanouil G. Spanakis

**Affiliations:** Computational Biomedicine Laboratory, Institute of Computer Science, Foundation for Research and Technology—Hellas, Heraklion, GR-70013 Crete, Greece; marspan@ics.forth.gr (M.S.); ssfak@ics.forth.gr (S.S.); sakkalis@ics.forth.gr (V.S.)

**Keywords:** complementary medicines, drug–herb interactions, eHealth, patient empowerment, personal health systems, pharmaceutical care

## Abstract

Herbal medicinal products (HMPs) are the subject of increasing interest regarding their benefits for health. However, a serious concern is the potential appearance of clinically significant drug–herb interactions in patients. This work provides an overview of drug–herb interactions and an evaluation of their clinical significance. We discuss how personalized health services and mobile health applications can utilize tools that provide essential information to patients to avoid drug–HMP interactions. There is a specific mention to PharmActa, a dedicated mobile app for personalized pharmaceutical care with information regarding drug–HMPs interactions. Several studies over the years have shown that for some HMPs, the potential to present clinically significant interactions is evident, especially for many of the top selling HMPs. Towards that, PharmActa presents how we can improve the way that information regarding potential drug–herb interactions can be disseminated to the public. The utilization of technologies focusing on medical information and context awareness introduce a new era in healthcare. The exploitation of eHealth tools and pervasive mobile monitoring technologies in the case of HMPs will allow the citizens to be informed and avoid potential drug–HMPs interactions enhancing the effectiveness and ensuring safety for HMPs.

## 1. Introduction

Medicinal plants and the scientific field of pharmacognosy always played an important role for discovering new chemical molecules with pharmacological actions that could be used as novel therapeutic drugs. Over the centuries, the use of products derived from plants or other natural sources represents an essential aspect in traditional medicine (e.g., Ayurveda and Chinese medicines) and society’s cultural aspects and practices in healthcare [1,2,3]. Over the last few decades, there has been a remarkable increase in popularity of health foods (nutraceuticals) and herbal medicinal products (HMPs) intake, and now hold a big share on the market of healthy nutrition and dietary supplements products [4,5,6]. There is also a growing interest on traditional Chinese medicine (TCM) which is based on HMPs along with body and mind practices (e.g., acupuncture) to improve healthcare. Such practices under different names are widely spread over China and East Asia (Japan and Korea), and continuously gain popularity in Europe and the USA [7,8,9,10,11,12,13]. Particularly for Europe (mainly northern countries) and the USA, phytotherapy (i.e., herbal medicine) represents a separate therapeutic approach based on the traditional use of plants for medical reasons. Phytotherapy utilizes two different approaches, namely (i) “rational phytotherapy” that refers to HMPs with documented efficacy and safety based on pharmacological and clinical data; and (ii) “traditional phytotherapy” which refers to products that their efficacy and safety has not yet been adequately described but their longitudinal use over the years has introduced them in the daily diet (i.e., tea consumption) [14,15].

**Figure 1:**
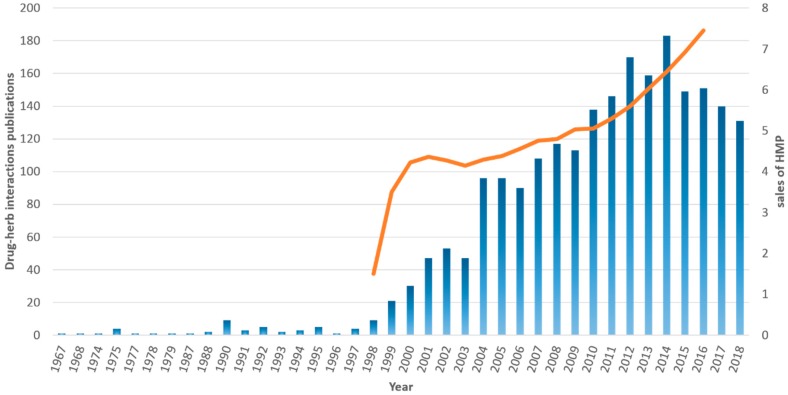
Trend of sales for herbal medicinal products (HMPs, orange line, based on data available in Ref. [6]) along with the number of publications available in PubMed referring to drug–HMP interactions (blue bars). A similar trend among total sales of HMP products and the number of scientific reports regarding drug–HMP interactions is evident revealing the increased concern of the scientific community on the matter.

To date, citizens’ and healthcare providers’ understanding regarding the usage of HMPs is still unclear with a lack of communication between them especially when it comes to the use of HMPs from a patient [16,17]. It is estimated that one out of two patients with a chronic disease will consume an HMP to improve their welfare, often without consulting a physician or a pharmacist [17,18,19,20]. The public’s opinion of HMPs is mainly influenced from perspectives mainly attributed to “traditional phytotherapy” practices, while at the same time marketing of HMPs still holds on the principle that the natural origin of these products marks them as safe to use. Public interest in HMPs can be attributed to several factors including: (i) public’s movement toward self-medication and trust on information that is exchanged between family and friends or sociological factors and beliefs that portray HMPs as more effective treatments mostly based on intuition rather than scientific data and reasoning; (ii) preference and invalid beliefs that natural-derived alternative medicines are superior to manufactured products; (iii) dissatisfaction with the outcomes from conventional treatment; (iv) high cost of novel medicines; (v) improvements in the manufacturing quality of HMPs; and (vi) mistrust on physician’s expertise to proper diagnose a health issue, as well as lack of communication that results in poor adherence and compliance with physician’s medical advice [21,22]. However, problems associated with the use of HMPs arise mainly from the classification of many of these products as foods or dietary supplements, thus reducing the required data from regulatory bodies regarding evidence of quality, efficacy, and safety prior to marketing authorization [23,24]. Studies thus far have revealed a number of cases that the use of HMPs and other dietary supplements can lead to potential and clinically significant interactions or clinical complications related to one or more of the ingredients of an HMP [20,25,26,27,28,29,30,31,32]. Interestingly, despite the fact that scientific reports on potential interactions between drugs and food products dates more than 40 years ago (in the last 20 years there has been a burst on scientific studies on potential drug–HMP interactions), there is still need to improve the dissemination of this information to general public (Figure 1). Considering the regulatory point of view, both FDA (https://www.fda.gov/) and EMA (https://www.ema.europa.eu/) have published relevant guidelines and directives describing the efficacy and safety criteria that a medicinal product of natural origin (apart of novel drugs) should meet in order to receive market authorization as botanical (FDA) or herbal medicinal product (EMA) for human use [33,34]. Generally, the FDA and EMA are in line with WHO guidelines that define HMPs as labeled products containing active ingredients obtained from the aerial or underground parts of botanicals or other plant materials or their combination [35,36]. These documents try to establish the regulatory processes needed to secure the quality and efficacy of HMPs but also to provide a roadmap of how potential implications of the simultaneous use of HMPs with conventional medicines can be predicted and avoided.

Today, modern healthcare aims to develop technologies on personal health systems focusing on innovative personalized health services and tools that empower individuals in well-being, disease prevention, optimum disease management, and provide recommendation services for the patient or the informal healthcare provider [37,38,39,40,41,42,43]. In this respect, an important aspect is the development of services that provide essential information regarding potential complications between prescribed medication, dietary supplement consumption (such as HMPs) or over-the-counter (OTC) use of drugs and HMPs [44,45,46]. This work aims to describe how personalized health services and mobile health applications can utilize tools that provide essential information to consumers and healthcare providers for managing potential drug-HMP interactions. In this regard, we also present PharmActa, a user friendly mobile app for personalized pharmaceutical care.

## 2. Materials and Methods

The clinical significance of drug interactions is an important aspect in clinical practice, as well as in research and development processes [47]. Drug interactions refer to the modulation of pharmacological effect of simultaneously administered medications, food, HMPs or dietary supplements [48]. The underlying pharmacological mechanisms of drug–HMP interactions can be related either to the modulation of pharmacokinetic (PK) processes (absorption, distribution, metabolism and elimination, ADME) or to pharmacodynamic (PD) mechanisms for interacting molecules due to synergistic or competitive effects in the site of action or in secondary biological targets [26,49,50]. In general, for PD related interactions, the biological associated factors for a drug are mostly associated with a low or narrow therapeutic index window or a variety of pharmacological actions in different biological targets. In PK related interactions, the biological factors are mostly associated with processes of drug absorption and metabolism due to inhibition or induction of (i) metabolic enzymes, such as the cytochrome P450 (CYP) and the uridine diphosphate-glucuronosyltransferase (UGT) conjugating enzymes, or (ii) transport proteins, such as the adenosine triphosphate–binding cassette (ABC) drug uptake/efflux transporters and the organic anion- transporting polypeptide (OATP) [51]. Inhibition or induction of these proteins may result in changes of drug concentration in the body above or below the therapeutic concentrations window that increase the risk for side effects or adverse drug reactions (ADRs) or lead in sub-therapeutic levels, respectively [26,52,53].

**Figure 2:**
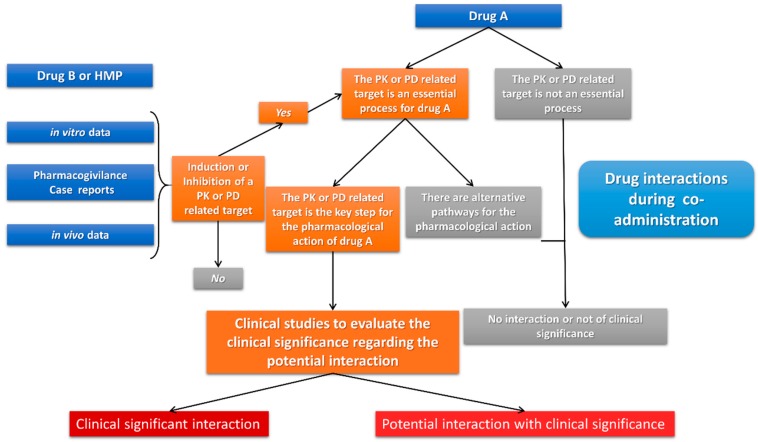
The evaluation process for estimating the clinical significance of a drug interaction between drug A and another medicinal product (drug B or HMP) according to FDA (based on Ref. [47]).

The methodological approaches to study a potential drug interaction are described in relative guidance provided by regulatory bodies (FDA, EMA) regarding their clinical significance (Figure 2) [47]. For example, in the case of PK drug–drug interactions, where one drug inhibits the metabolic pathway of another, based on the fold increase of the PK parameter, the interaction can be categorized as minor, moderate, or major regarding its clinical significance [47,51]. Following similar approaches, in vitro, in vivo, and clinical studies thus far highlight the existence of a number of clinically significant drug–HMP interactions, some of which are even included in relative information tables by regulatory bodies (i.e., FDA guidance for industry) [47].

The most well-known example of drug–HMP interaction in scientific community is St. John’s Wort (SJW) interactions with other drugs. SJW has been described through several studies as an HMP product that can lead in clinically significant PK and PD interactions [54,55,56]. Especially for SJW, the underlying pharmacological mechanisms of the PK and PD interactions have been described in detail [57,58]. SJW contain different groups of compounds such as hypericin, hyperforin, and flavonoids. Hyperforin in initially administered doses of SJW have been shown to inhibit the activity of several CYP enzymes (i.e., 1A2, 2C9, 2C19, 2D6, and 3A4) which may lead to increased drug exposure for drugs metabolized from those enzymes, whereas in long term use of SJW, hyperforin mediates the induction of expression levels for several CYPs and for P-glucoprotein (P-gp, an ABC efflux transporter) and thus lowering the concentrations of drugs in blood circulation leading to clinical significant drug–HMP interactions due to increased metabolic clearance (where drugs reach subtherapeutic levels) [58,59]. In addition, it has been proposed that the PD mechanism of interaction between drugs and SJW is attributed to the elevated levels of serotonin when combined with antidepressants and thus increasing the risk for induced serotonin syndrome [60].

However, for the majority of HMPs the results are sometimes vague or contradictory regarding the clinical significance of drug–HMP interactions compared to typical drug–drug interactions studies. The results for drug–HMP interactions posing mostly a potential to present an interaction rather than a clear biological mechanism [49,61]. This can be attributed on the fact that the HMPs are mixtures of many chemical constituents and/or plant materials, standardized based on one or more representative chemical molecules of the herb and thus they often exhibit variability in their formulations that impacts also the results from studies focusing on potential drug–HMP interactions. As an example, in the case of SJW, studies have demonstrated that when different SJW products with variable content in hyperforin are used, the outcome of interaction changes [62]. In addition, limited data are available regarding the PK properties of HMPs constituents in order to determine specific PK parameters, such as bioavailability and blood concentrations of a compound in a given dose. Furthermore, there are limited data available that clarify the pharmacological mechanism involved, whereas there are a lot of clinical studies focusing on the therapeutic outcome of the use of an HMP product [61,63]. This adds to the burden in the exploitation of in vitro/in silico and in vivo data at clinical level and in the design of clinical studies that attempt to address possible pharmacological mechanisms involved, as well as the clinical significance of a potential drug–HMP interaction [62]. In this regard, in cases that clinical studies are not available, the assessment for potential drug–HMP interactions is approached indirectly through combination of data from several sources such as in vitro, in silico or in vivo findings, the clinical importance of which remains to be evaluated. Moreover, data from case report studies, in which causality can be assessed, usually are applied in order to suggest caution and consideration prior to the use of a HMP.

The potential drug–HMP interactions can be classified based on the availability of scientific data regarding the pharmacological outcome that should be considered for the optimum management [64]. Drug–HMP interactions can be of a minimum effect up to cases where combination should be avoided due to high risk of ADRs, treatment failure or toxicity. To this respect, the clinical significance of potential drug–HMP interaction can be categorized as: (A) “the HMP should be avoided and medical advice should be sought” in the case that the pharmacological mechanism and clinical data describe sufficiently the significance of the interaction; (B) “the HMP can be used only after medical advice” in the case that specific findings from experimental approaches and case reports suggest a potential causality for interaction; and (C) “the HMP can be used but it is in person benefit to inform his/her treating physician” in the case that the data are not available or limited. The proposed approach can allow the distribution of available information from scientific reports, to be delivered to the public in a comprehensive way avoiding scientific terms that are not well received or understood from the general public.

Nowadays, apart from the public’s interest in HMPs, there is a prevalent interest regarding health information that is supported by numerous medical oriented webpages, applications and social media. Among others there are several publicly available webpages where information about medicines can be retrieved regarding potential interactions. However, they are mainly medical-oriented databases, which subtracts from their usability from the general public. On the contrary, webpages that provide information regarding HMP seem to present them in a more simplified way. Adopting this challenge, Information and Communication Technologies (ICT) have allowed the generation of a variety of tools and applications for personal health data management that are able to provide personalized feedback and recommendation, through easily accessible and automated services, for the patient or informal caregiver [42,65]. Typical examples of such tools and applications are the development of services that allow end-users to manage and update their personal health data [38,40,66]. These applications have also encompassed tools regarding drug–drug and drug–HMP interactions that exploit available databases and provide feedback in simple and comprehensive way [46].

In the context of tools and applications for HMP, there are also several systems designed and implemented focusing on potential drug–herb interactions [67,68]. These systems are medically oriented in an effort to provide necessary information to the medical personnel regarding potential interactions for HMPs, especially for products that their use is evident among the public. Moreover, there is a growing need to develop services for special population groups, such as cancer patients, which seem to use HMPs during chemotherapeutic scheme periods [58,69,70]. These services are implemented and mainly driven by the fact that there is a lack of communication between the treating physicians, medical personnel (pharmacists, nurses) and cancer patients with respect to the safety and efficacy of using HMP products. As a result, it is of great importance to develop tools and systems that provide necessary and personalized information for healthcare to citizens, including information regarding the safe use of HMP products. Recently, we have demonstrated how issues of drug interactions involving drugs, HMPs and food can be addressed through personalized empowerment services for healthcare [46]. Furthermore, we have demonstrated how web-based systems for the management of drug interactions with complementary and alternative medicines can be designed and developed [71]. These approaches take into consideration that when suitable data are available, they can be efficiently incorporated for the generation of user-friendly services that empower patients providing personalized information in response to user’s request and provide quick and secure personalized feedback with respect to the administered treatment. This approach has allowed us to design and deliver a mobile application for personalized pharmaceutical care for the people (PharmActa).

## 3. Results

**Figure 3:**
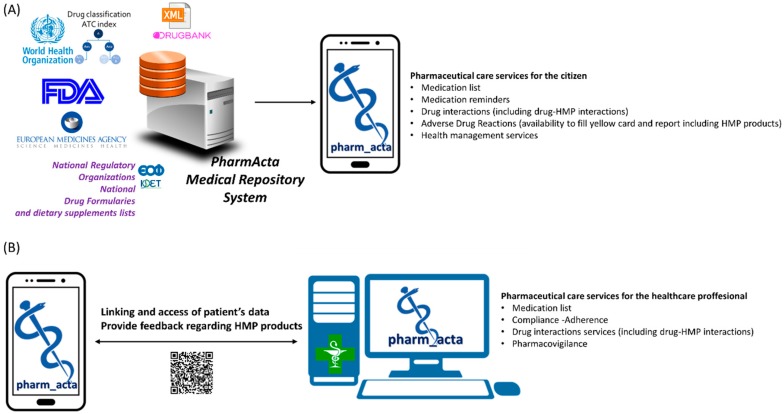
(**A**) Indicative data sources that integrate into PharmActa repository for the generation of the mobile app (including the Greek National Formulary from the Greek National Organization for Medicines, www.eof.gr). (**B**) Representation of PharmActa mobile app link with healthcare provider systems (PharmActa’s specialized view for healthcare providers generates a QR code that is readable from patient’s mobile camera who subsequently grants temporarily access to medical data).

PharmActa is developed using the available databases from WHO (ATC) (https://www.who.int/medicines/regulation/medicines-safety/toolkit_atc/en/), Drugbank [72] and FDA combined with scientific information from relevant data sources (Medline). The design of the drug information repository follows the ISO Identification of Medicinal Products (IDMP) set of standards that have been adopted by the European Union and the EMA, as the means for the unique identification of medicinal products and the standardization of the associated information [73]. The core of the backend infrastructure is the PharmActa Knowledge Base (KB) that integrates databases of drug- and HMP- related information, standardized terminologies, and information about the pharmaceutical products in use. This comprehensive repository is accessible through a service layer that provides its content to the end-user applications, i.e., the mobile application and the web-based application for medical professionals [71]. The PharmActa mobile application serves as an interconnection point between consumer and healthcare provider (Figure 3).

The severity of the potential drug–HMP interactions in PharmActa, is classified taking into consideration available information found in other databases—such as Medline, Medscape (https://reference.medscape.com/drug-interactionchecker), drugs.com (https://www.drugs.com/drug_interactions.html), WebMD (https://www.webmd.com/interaction-checker/default.htm), RxList (https://www.rxlist.com/drug-interaction-checker.htm)—and classified according to their clinical significance based in the availability of well-established data from scientific studies throughout literature search. The potential drug–HMPs can be classified based on the severity of the pharmacological outcome, which should be considered for the optimum management. Generally, the drug–HMP interactions can be of a minimum effect up to cases where combination should be avoided due to high risk of ADRs, treatment failure or toxicity. In this respect, it can be stated that the clinical significance of potential drug–HMP interaction can be categorized in three easy to follow levels (Table 1).

Scientific studies over the previous years have found several HMP products to be responsible for potential drug–herb interactions. Apart of SJW, several other works have focused on a number of cases of HMPs that have been reviewed and presented in a series of other scientific studies [20,27,28,29,30,31,32,61]. In this work, we present a different approach, where we take into consideration data regarding the top selling HMPs according to recently published reports for 2017 [6]. For these HMP products, a research through Medline was implemented regarding potential drug–herb interactions data (in vitro/in silico/in vivo and clinical). The evaluation of the available data was implemented through assessment of five levels of existing data: (i) potential interactions based on theoretical data and the suggested mechanism of action for the HMP product, (ii) in vitro/in vivo assessment of uncharacterized extracts, (iii) in vitro assessment of characterized extracts, (iv) in vivo assessment of characterized extracts, and (v) in vitro/in vivo/clinical assessment of characterized extracts [20,49,74]. The list of the HMP is adopted from the report of Smith et al. based on the information regarding retail sales in the USA [6]. Overall, the list includes 40 HMPs with several indications of use with the most frequent to be potentially antioxidant, antidiabetic, and antilipidemic products. Out of the 40 HMPs, available data are found for the 32 of them (80%) regarding their potential to present drug–HMP interactions. In addition, approximately 32% of the identified interactions seem to be related with PK processes, 51% to be related with PD mechanisms, while the rest (17%) is related to interactions implicating both PK and PD processes. Moreover, the severity of interactions revealed that for 9% of the HMPs, the co-administration should be avoided and a medical advice should be sought, for 44% of them the HMP product could be combined only under medical advice and specific circumstances (precautions should be met to minimize any risk), and the rest 47% can be used but always in accordance with the treating physician. Table 2 summarizes the data of the HMP products along with the proposed use; drugs that may lead to drug–HMP interactions; the potential pharmacological mechanism; and how these data are integrated and presented in the PharmActa mobile app.

## 4. Discussion

This work aimed to provide a brief overview regarding drug–HMPs interactions and how the evaluation of their clinical significance can be disseminated in order to be available to the general public through personalized health services and mobile health applications. Although there are a lot of studies focusing on the subject of drug–HMP interactions and even if the pharmacological mechanisms are thoroughly clarified in some cases, the public’s opinion seems to remain the same, while the use of HMP products is still increasing. At the same time there seems to be a lack of communication for providing information between medical personnel and patients towards patient empowerment and advanced pharmaceutical care [16,17,18,101].

PharmActa implementation is based on the integration of publicly available databases that had to be assembled, annotated, and validated manually. Our implementation supports data entry, data export, and database annotation while complying with standards for manual data validation [46,71]. PharmActa user interface is tested against “supporting needs” for the users in order for the first version of the tool to be available for pharmacists and public. PharmActa validation and market penetration is still an ongoing process towards its successful commercial adoption. The drug repository of PharmActa incorporates among others, data for many HMP products. In this work, we focused on the products with increased sales during the previous years in western countries (US) [6].

For most of these HMPs sufficient data for potential interactions exist which should be able to be disseminated among patients towards patient empowerment and advanced pharmaceutical care. In this respect, the use of a mobile app such as PharmActa from patients and healthcare providers (i.e., pharmacists) would allow the retrieval of information regarding the safety of using an HMP from that list along with a co-administered medication. For the majority of the presented HMPs the interactions are related with medications for chronic diseases, such as cardiovascular disorders (CVD) and other related problems (clot disorders, heart failure), diabetes, cancer, arthritis, and transplant patients, which is in line with recently published data [27,29,49]. The drugs in these categories are mostly of narrow therapeutic index and with high probability for presenting ADRs, which may lead in additional health complications (https://www.fda.gov/Drugs/DevelopmentApprovalProcess/DevelopmentResources/DrugInteractionsLabeling/ucm110632.htm) [102]. Moreover, there seems to be possible pairing between the proposed uses for the HMP, the disease a patient may have, and the data for interactions. To this respect, the need to further empower patients and healthcare providers to ensure the safety and efficacy of HMPs in promoting health status, becomes evident.

The assessment of ICT technologies today is essential to design and promote tools ensuring compliance and adherence with the administered treatment. This is in line with the increased interest from the general public to seek information regarding their health status, as well as to seek alternative treatments and products for the management of a chronic disease [103,104]. At the same time healthcare providers, in order to keep patients updated with modern healthcare, try to provide solutions regarding patient empowerment assuming a more active role for their patients. Generally, advanced pharmaceutical care services for avoidance of drug-related problems can have an important impact optimizing disease management and treatment outcome. Pharmacists in community pharmacy stores or in clinics are usually the most readily assessable members in modern healthcare. By updating and adopting a more active involvement as healthcare providers, they can play an important role in modern healthcare. The utilization of their knowledge and expertise especially for monitoring drug interactions and notifying the physician and patients about potential problems can be beneficial towards optimization of administered treatments [16,17,18,26,44,68,101,105,106]. In this respect, a feasible way is the promotion of ICT eHealth tools and applications that assist patients to be compliant and adherent with their prescribed medication while at the same time providing them with necessary and personalized information for the management of their personal health data [40,41,42,43,68,71]. PharmActa enhances the communication representing connection link between pharmacists or other healthcare providers and patients, while at the same time providing a tool for the user to seek and understand related problems that may occur during their administered therapy.

## Figures and Tables

**Table 1:** Clinical significance of drug–HMP interactions as they are characterized in PharmActa (adopted from Refs. [46,71]).

Severity	Clinical Guidance	Presentation in PharmActa
Prohibited	Use alternative-Major interaction -Avoid co-administration	Avoid it and seek medical advice
It should be avoided	Contraindicated-Major interaction-Combine only under specific circumstances	
Minimize the risk	Use with caution-Moderate interaction-Precautions to minimize any risk	Use it only after medical advice
No clinical impact	Minor interaction-research data suggest no clinical impact	Use it but always inform your treating physician
No interactions	Clinical /research results suggest that there is no interaction	

**Table 2:** Top selling HMPs and how available data are presented in PharmActa regarding potential drug–herb interactions.

HMP	Proposed Use	Potential Drug Interactions	Pharmacological Mechanisms	Patients-Diseases	Clinical Significance	PharmActa	Reference
**Horehound**	respiratory ailments	-	-	-	-	monitor for clinical significant DHIs	-
**Cranberry**	bladder and kidney ailments	anticoagulant and antiplatelet therapy	PK and PD	CVD and clot disorders	Research level-clinical data suggest no interaction	use with caution	[75]
**Echinacea**	immune stimulant	antipsychotics, antidepressants, immunosuppressants	PK and PD	CNS disorders	Under specific circumstances-Precautions to minimize any risk	monitor for clinical significant DHIs	[17,26,27,28,61]
**Green Tea**	antioxidant	anticoagulant and antiplatelet therapy	PD	CVD and clot disorders	Research level-clinical data suggest no interaction	use with caution	[76]
**Black Cohosh**	PMS and menopause symptoms	amiodarone, fexofenadine, glyburide, simvastatin, atorvastatin	PK	CVD and clot disorders	Research level-clinical data suggest no interaction	use with caution	[26,27,51,58]
**Garcinia**	weight loss	ciprofloxacin, quinine	PK	bacterial infections and muscular disorders	Research level-clinical data suggest no interaction	use with caution	[25]
**Flax Seed/Flax oil**	CVD problems and diabetes	anticoagulant and antiplatelet therapy	PD	CVD and clot disorders	Research level-clinical data suggest no interaction	use with caution	[77]
**Ginger**	reduce nausea and inflammation	tacrolimus	PK	allogeneic organ transplant	Under specific circumstances-Precautions to minimize any risk	monitor for clinical significant DHIs	[32,58,61,78]
**Ivy leaf**	expectorant	-	-	-	Research level-clinical data suggest no interaction	use with caution	[32,79]
**Turmeric**	antiinflamantory	CVD drugs, antidepressants, anticoagulants, antibiotics, chemotherapeutic agents, and antihistamines	PK	CVD and clot disorders, CNS disorders, Cancer, bacterial infections, allergies	Under specific circumstances-Precautions to minimize any risk	monitor for clinical significant DHIs	[32,80]
**Valerian**	anxiety and insomnia	benzodiazepines and sedatives	PD		Under specific circumstances-Precautions to minimize any risk	monitor for clinical significant DHIs	[26,27,81]
**Fenugreek**	diabetes and PMS	hypoglycaemic, anticoagulant, and antiplatelet therapy	PD	diabetes, CVD and clot disorders	research level-clinical data suggest no interaction	use with caution	[82]
**Yohimbe**	erectile dysfunction - weight loss	MAOIs, clonidine,	PD	CNS disorders, prostate hypertrophy, kidney disease	avoid co-administration	use alternative due to clinical significant DHIs	[20]
**Aloe**	constipation, diabetes, acne and inflamation	digoxin, antidiabetic drugs	PD	HF, diabetes	under specific circumstances-precautions to minimize any risk	monitor for clinically significant DHIs	[61,83]
**Saw Palmeto**	prostate surgery	antibiotics, anticoagulant, and antiplatelet therapy	PD	CVD and clot disorders, bacterial infections,	research level-clinical data suggest no interaction	use with caution	[26,27,28,84]
**Milk Thistle**	liver problems	losartan, warfarin, phenytoin, diazepam	PK	CVD and preoperative period	research level-clinical data suggest no interaction	use with caution	[26,51,85]
**Garlic**	reducing cholesterol and blood pressure	colchicine, digoxin, doxorubicin, qunidine, rosuvastatin, dexamethasone, nifedipine, verapamil, saquinavir, indinavir, ritonavir, rifampicin, reserpine, carbamazepine	PK	arthritis, CVD, cancer, HIV	under specific circumstances-precautions to minimize any risk	monitor for clinically significant DHIs	[26,28,32,51,83,86]
**Coconut Oil**	eczema and improve HDL cholesterol levels	-	-	-	-	use with caution	-
**Eldeberry**	Constipation and flu symptoms	immunosuprresants, hypoglycemic	PD	transplantation, diabetes	research level-clinical data suggest no interaction	use with caution	[78]
**Cinnamon**	diabetes and lowering cholesterol	anticoagulant and antiplatelet therapy	PD	CVD and clot disorders	research level-clinical data suggest no interaction	use with caution	[87]
**Green Coffee Extract**	antioxidant for diabetes and heart disease	adenosine, anticoagulant and antiplatelet therapy, MAOIs, CVD drugs	PD	CVD and clot disorders, CNS disorders, Inflammatory bowel disease	under specific circumstances-precautions to minimize any risk	monitor for clinical significant DHIs	[88]
**Boswellia**	anti-inflammatory in asthma and arthritis	-	-	-	-	monitor for clinical significant DHIs	-
**Ginkgo**	adaptogen for dementia and fatigue	clopidogrel, aspirin, warfarin	PD		under specific circumstances-precautions to minimize any risk	monitor for clinical significant DHIs	[20,26,28,30,51,61,85]
**Plant Sterols**	lower cholesterol levels	LLTs	PD	dyslipidemias	research level-clinical data suggest no interaction	use with caution	[89]
**Senna**	constipation	digoxin, warfarin	PD		avoid co-administration	use alternative due to clinical significant DHIs	[90]
**Acaí**	antioxidant	-	-	-	-	-	
**Guarana**	CNS stimulant	adenosine, anticoagulant and antiplatelet therapy, MAOIs, CNS drugs	PD	CVD and clot disorders, CNS disorders	under specific circumstances-precautions to minimize any risk	monitor for clinically significant DHIs	[91]
**Rhodiola**	adaptogen	loasartan, warfarin, clopidogrel	PK	CVD and clot disorders	under specific circumstances-precautions to minimize any risk	monitor for clinically significant DHIs	[92,93,94,95,96,97]
**Bioflavonoid Complex**	antioxidant	digoxin, anticoagulant and antiplatelet therapy, CVD, cancer medications	PK	CVD and clot disorders, Cancer	moderate	monitor for clinical significant DHIs	[85]
**Red Yeast rice**	lower cholesterol levels	LLTs, CVD drugs	PK and PD	dyslipidemias, CVD	under specific circumstances-precautions to minimize any risk	monitor for clinically significant DHIs	[92,96]
**Ginseng (siberian)**	adaptogen and heart disease	digoxin	PK	heart failure	under specific circumstances-precautions to minimize any risk	monitor for clinically significant DHIs	[26,28,30,51,61,68]
**Horny Goat Weed**	erectile dysfunction, PMS, osteoporosis	CVD, anticoagulant and antiplatelet therapy	PD	CVD and clot disorders	research level-clinical data suggest no interaction	use with caution	[98]
**Yerba Mate**	CNS stimulant	adenosine, anticoagulant and antiplatelet therapy, MAOIs, CNS drugs	PD	CVD and clot disorders, CNS disorders	research level-clinical data suggest no interaction	use with caution	-
**Fennel**	digestive problems, pregnancy	Contraceptives, tamoxifen, ciprofloxacin	PK	birth control, breast cancer, infections	under specific circumstances-precautions to minimize any risk	monitor for clinically significant DHIs	[78]
**Beta Glucans**	lower blood cholesterol	LLTs, CVD drugs	PK and PD	dyslipidemias, CVD	under specific circumstances-precautions to minimize any risk	monitor for clinically significant DHIs	[99]
**Maca**	female hormone imbalance, menstrual problems, chronic fatigue syndrome	-	-	-	-	-	-
**St. John’s Wort**	antidepressant	anticancer, CNS drugs, LLTs, PPIs, antiretroviral, hypoglycemic, antihistamine, CVD drugs, antimicrobials, hormonal agents, immunosuppressants	PK and PD	arthritis, CVD, cancer, CNS disorders, diabetes, HIV, infections, allergies, transplantation	avoid co-administration	use alternative due to clinical significant DHIs	[15,28,56,58,59,61,62]
**Wheatgrass/Barley**	diabetes, lower cholesterol and for weight loss	-	-	Inflammatory bowel disease	under specific circumstances-precautions to minimize any risk	monitor for clinical significant DHIs	[88]
**Goji Berry**	antioxidant, diabetes, blood pressure	warfarin and antiplatelet	PD	CVD and clot disorders	under specific circumstances-precautions to minimize any risk	monitor for clinical significant DHIs	[100]
**Chia Seed/Chia oil**	omega-3 fatty acids and antioxidants	-	-	-	-	-	

DHIs: Drug-Herb interactions; PK: Pharmacokinetics; PD: Pharmacodynamics; CVD: Cardio-Vascular Diseases; CNS: Central Nervous System; MAOIs: Monoamine Oxidase Inhibitors; HF: Heart Failure; HIV: Human Immunodeficiency Virus; HDL: High Density Lipoproteins; LLTs: Lipid Lowering Therapies; PPIs: Proton Pump Inhibitors; PMS: Premenstrual Syndrome.
